# Supplementary material for: sciLaMA: A Single-Cell Representation Learning Framework to Leverage Prior Knowledge from Large Language Models
Source: bioRxiv. 2025 May 29:2025.01.28.635153. Preprint. [Version 2] doi: 10.1101/2025.01.28.635153 (PMC12154950; doi:10.1101/2025.01.28.635153)
Supplement: Supplement 1 [file NIHPP2025.01.28.635153v2-supplement-1.pdf]

bioRxiv preprint doi: https://doi.org/10.1101/2025.01.28.635153; this version posted May 29, 2025. The copyright holder for this preprint (which was not certified by peer review) is the author/funder, who has granted bioRxiv a license to display the preprint in perpetuity. It is made available under aCC-BY 4.0 International license.

Appendix
sciLaMA: A Single-Cell Representation Learning Framework to Leverage Prior Knowledge from Large Language Models

A. Model Input Processing:

A.1. Cell Encoder Input

As mentioned in **Section 3 Methods**, the input for cell Encoder is the scRNA-seq data for specific cell population $\boldsymbol{c}$. Therefore, $\boldsymbol{c}_{i,j}$ denotes the scaled log-normalized expression value of gene $\boldsymbol{j}$ in cell $\boldsymbol{i}$.

The raw scRNA-seq expression matrix, $\boldsymbol{c}^{\text{raw}}$, is a sparse count matrix. For use in sciLaMA, the data after quality control (QC) is processed through library size normalization and feature-wise z-score scaling to achieve zero mean and unit variance. Values beyond ±10 are clipped. Specifically, the normalized expression $\boldsymbol{c}^{\text{norm}}$ is calculated as:

$$\boldsymbol{c}_{i,j}^{\text{norm}} = \log_e \left(1 + 10^4 \times \frac{\boldsymbol{c}_{i,j}^{\text{raw}}}{\sum_{k=1}^{m} \boldsymbol{c}_{i,k}^{\text{raw}}} \right)$$

Here, $\boldsymbol{c}_{i,j}^{\text{raw}}$ represents the raw count value of gene $\boldsymbol{j}$ in cell $\boldsymbol{i}$, and $\sum_{k=1}^{m} \boldsymbol{c}_{i,k}^{\text{raw}}$ is the total expression counts number for cell $\boldsymbol{i}$. The multiplication by 10^4 ensures a standardized size factor for normalization. This normalization procedure adjusts for library size differences across cells and prepares the data for following analysis.

A.2. Gene Encoder Input

In this study, sciLaMA integrated static gene embeddings from six external sources across three distinct modalities.

Source	Dimensionality	Modality
ChatGPT	1536	Text
GenePT (NCBI)	1536	Text
ESM	5120	Protein Sequence
ProtTrans	1024	Protein Sequence
scGPT	512	Single Cell
CellPLM	1024	Single Cell

Table S1. Gene Embedding Sources and Characteristics.

A.2.1 Natural Language Embeddings

We acquired text description-based gene embeddings from two studies: GenePT and scELMo (Chen & Zou, 2024; Liu et al., 2023), utilizing the OpenAI text-embedding-ada-002 model (OpenAI, 2022). These embeddings were generated using two distinct text corpora: GPT-3.5 generated descriptions (referred to as ChatGPT) and National Center for Biotechnology Information (NCBI) gene card summaries (referred to as GenePT). We obtained 1,536-dimensional static embeddings for each gene (d = 1,536).

A.2.2 Protein Language Embeddings

We derived protein sequence-based gene embeddings from two protein language models: ESM2 t48_15B_UR50D with 5,120-dimensional embeddings per gene (Lin et al., 2023), and ProtXLNet from ProtTrans with 1,024-dimensional embeddings (Elnaggar et al., 2022) from the SATURN study (Rosen et al., 2024). These embeddings were generated using the amino acid sequences of each corresponding gene.

A.2.3 Single-Cell Gene Language Embeddings

bioRxiv preprint doi: https://doi.org/10.1101/2025.01.28.635153; this version posted May 29, 2025. The copyright holder for this preprint (which was not certified by peer review) is the author/funder, who has granted bioRxiv a license to display the preprint in perpetuity. It is made available under aCC-BY 4.0 International license.

For single-cell foundation models, we retrieved static gene embeddings from two pretrained models: scGPT-whole-human (512-dimensional embeddings) (Cui et al., 2024) and cellPLM (1,024-dimensional embeddings) (Wen et al., 2023). The scGPT embeddings were obtained using the model's GitHub tutorial, while cellPLM embeddings were extracted from the embedder module's feature encoder parameters, as directed by the authors.

B. Model Optimization Illustration:

The sciLaMA model optimization process, comprehensively described in **Section 3 Methods**, is illustrated through a stepwise training strategy visualization (Figure S1). The optimal hyperparameter values chosen for our experiments are scalar $\gamma = 0.05$ and latent dimensionality $K = 40$, based on the evaluations presented in Table S8 and Table S9.

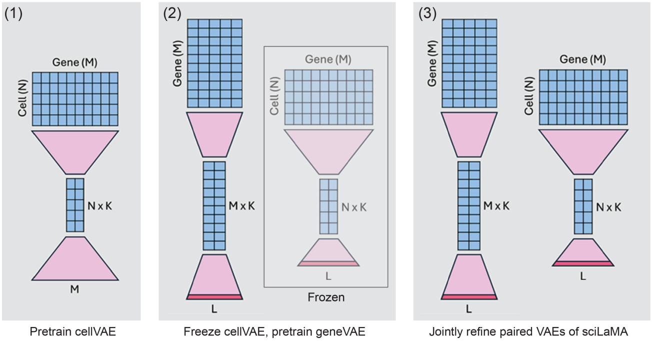

Figure S1. Schematic representation of the progressive optimization workflow for the sciLaMA framework. (Box indicates freezing parameters.)

C. Dataset Introduction:

C.1. Experiment 1: Cell Representation Learning Benchmarking

This experiment benchmarks cell representation learning methods using a combination of single-cell RNA sequencing datasets derived from five studies focused on the pancreas. The data includes a total of 14,767 cells spanning 13,062 genes (after intersection with precomputed static gene embeddings).

Datasets Used: Baron et al.: 8,569 cells (Baron et al., 2016); Segerstolpe et al.: 2,127 cells (Segerstolpe et al., 2016); Muraro et al.: 2,122 cells (Muraro et al., 2016); Xin et al.: 1,492 cells (Xin et al., 2016); Wang et al.: 457 cells (Wang et al., 2016).

The aggregated dataset was a gold-standard benchmarking dataset originally analyzed in the context of batch-effect correction, as described in Tran et al., in 2020 (Tran et al., 2020). This benchmarking experiment evaluates the performance of cell representation learning in mitigating batch effects while preserving biological signal.

C.2. Experiment 2: Gene Expression Imputation Benchmarking

This experiment evaluates the accuracy of gene expression imputation approaches by leveraging two complementary datasets (Zeisel et al., 2015; Codeluppi et al., 2018):

Reference scRNA-seq Dataset (Zeisel et al., 2015):

· Number of cells: 3,005

· Total genes: 19,972, with 3,654 highly variable genes selected for benchmarking.

· Validation: A 10% validation split is used for early stopping during model training.

Spatial Transcriptomics Dataset (Codeluppi et al., 2018):

· Number of spatial spots: 4,530

bioRxiv preprint doi: https://doi.org/10.1101/2025.01.28.635153; this version posted May 29, 2025. The copyright holder for this preprint (which was not certified by peer review) is the author/funder, who has granted bioRxiv a license to display the preprint in perpetuity. It is made available under aCC-BY 4.0 International license.

· Genes: 30, analyzed using a leave-one-gene-out approach to simulate imputation scenarios.

This setup allows for assessing the generalizability of gene imputation models.

C.3. Experiment 3: Marker Gene Identification

This experiment focuses on identifying marker genes for distinct cell types using the human Peripheral Blood Mononuclear Cell (PBMC) 3K dataset from 10x Genomics, a legacy dataset widely utilized in tools like Seurat (Butler et al., 2018) and scanpy (Wolf et al., 2018) tutorials. The ground truth gene markers and cell type annotations were obtained from the tutorials (`https://satijalab.org/seurat/articles/pbmc3k_tutorial`, and `https://scanpy.readthedocs.io/en/stable/tutorials/basics/clustering-2017.html`).

Dataset Details:

· Initial Size: 2,700 cells × 32,738 genes

· Final Size: 2,700 cells × 9,540 genes (post-filtering and intersection with static gene embeddings).

Cell Type	Number of cells
CD4 T cells	1158
CD14 Monocytes	487
B cells	357
CD8 T cells	329
FCGR3A Monocytes	160
NK cells	160
Dendritic cells	36
Megakaryocytes	13

Table S2. Cell Type Statistics of human PBMC 3K dataset

C.4. Experiment 4: Trajectory Analysis and Temporal Dynamic Gene Discovery

This experiment investigates gene dynamics along developmental trajectories using the P0 mouse cortex dataset from the SNARE-seq study (Chen et al., 2019). The original SNARE-seq dataset includes both transcriptomic and epigenomic information from the same single cells, but we only utilized the transcriptomic data with 1,469 cells and 8,293 genes (after intersection with precomputed static gene embeddings). This experiment focuses on uncovering temporally dynamic genes critical for neurodevelopmental processes. The ground truth gene markers and cell type annotations were obtained from the original study.

Cell Type	Number of cells
IP_Hmgn2	214
IP_Gadd45g	99
IP_Eomes	437
Ex23_Cntn2	177
Ex23_Cux1	542

Table S3. Cell Type Statistics of mouse P0 cortex dataset

D. Benchmarking Metrics Introduction:

To comprehensively evaluate the performance of various methods, we employ metrics tailored to different aspects of single-cell data analysis, including cluster annotation accuracy, cell type separation, batch mixing quality, and predictive/imputation accuracy (Li et al., 2022; Luecken et al., 2022), briefly summarized below:

D.1. Clustering and Annotation Accuracy

To assess the biological relevance of clustering and annotation based on the learned embeddings, we employ:

bioRxiv preprint doi: https://doi.org/10.1101/2025.01.28.635153; this version posted May 29, 2025. The copyright holder for this preprint (which was not certified by peer review) is the author/funder, who has granted bioRxiv a license to display the preprint in perpetuity. It is made available under aCC-BY 4.0 International license.

· **Adjusted Rand Index (ARI):** Measures the agreement between predicted and ground-truth cluster labels, adjusted for chance. A higher ARI indicates better alignment between predicted clusters and original biological annotations, reflecting more accurate and biologically meaningful clustering.

· **Normalized Mutual Information (NMI):** Quantifies the mutual dependence between predicted clusters and ground-truth cell type annotation labels, normalized to account for the total number of clusters. A higher NMI indicates better clustering accuracy.

D.2. Cell Type Separation

To evaluate how well methods preserve separation between distinct cell types, we employ:

· **Average Silhouette Width (ASW):** Evaluates the cohesion within clusters and the separation between them. Higher ASW scores indicate that cells within the same cluster are more similar to each other than to cells in other clusters, signifying well-defined clusters.

· **Graph Cell-Type Integration Local Inverse Simpson's Index (cLISI):** Measures the local diversity of cell types within neighborhoods in an integrated graph representation. High cLISI values suggest better grouping of similar cell types in the embedding space.

D.3. Batch-Effect Correction Quality

To evaluate batch effect removal while preserving biological variance, we apply:

· **Batch-Adjusted Silhouette Width (batchASW):** Evaluates the extent of batch mixing while penalizing over-mixing of unrelated cells. Higher batchASW scores indicate better batch integration without compromising biological separation.

· **Graph Integration Local Inverse Simpson's Index (iLISI):** Measures the diversity of batch labels within local neighborhoods of an integrated graph. Higher iLISI scores indicate more uniform batch mixing, reflecting better integration while preserving cell type integrity.

D.4. Predictive Accuracy and Divergence Metrics

For imputation and gene expression prediction tasks, we employ:

· **Pearson Correlation Coefficient (PCC):** Assesses linear relationships between predicted and observed gene expression values, with higher values indicating stronger correlations.

· **Spearman Correlation Coefficient (SCC):** Evaluates rank-based relationships, capturing monotonic correlations between predicted and observed values, providing insights into the consistency of expression patterns.

· **Jensen-Shannon Divergence (JSD):** Measures the similarity between predicted and true gene expression distributions. Lower JSD values indicate better agreement between the two distributions.

· **Root Mean Square Error (RMSE):** Quantifies the average magnitude of errors between predicted and observed values. Lower RMSE scores reflect higher accuracy

D.5. Clustering Quality Metrics To evaluate the geometric coherence and separation of clusters in the learned gene embedding space, we include two additional metrics:

· **Davies-Bouldin Index (DBI):** Quantifies the ratio of intra-cluster dispersion to inter-cluster separation. Lower DBI values indicate better-defined clusters with high intra-cluster similarity and distinct separation between clusters.

· **Calinski-Harabasz Score (CHS):** Measures the ratio of between-cluster dispersion to within-cluster dispersion. Higher CHS values reflect dense, well-separated clusters

E. Supplementary Results:

bioRxiv preprint doi: https://doi.org/10.1101/2025.01.28.635153; this version posted May 29, 2025. The copyright holder for this preprint (which was not certified by peer review) is the author/funder, who has granted bioRxiv a license to display the preprint in perpetuity. It is made available under aCC-BY 4.0 International license.

Methods	w\ external knowledge	ARI ↑	NMI ↑	ASW ↑	batchASW ↑	iLISI ↑
sciLaMA-GenePT	√	0.545	0.767	0.539	0.863	0.240
sciLaMA-CellPLM	√	0.479	0.723	0.541	0.871	0.257
sciLaMA-ProtTrans	√	0.547	0.749	0.538	0.864	0.229
sciLaMA-ChatGPT	√	0.545	0.762	0.534	0.863	0.225
sciLaMA-scGPT	√	0.522	0.746	0.526	0.867	0.223
sciLaMA-ESM	√	0.494	0.722	0.529	0.864	0.253
sciLaMA (s.i.)	×	0.436	0.698	0.539	0.832	0.210

Table S4. Cell representation learning and integration performance on human pancreatic datasets across variants of sciLaMA models

Methods	cLISI ↑
sciLaMA-GenePT	0.995
sciLaMA-CellPLM	0.995
sciLaMA-ProtTrans	0.992
sciLaMA-ChatGPT	0.993
sciLaMA- scGPT	0.993
sciLaMA- ESM	0.993
sciLaMA (s.i.)	0.987
scGPT fine-tuned	0.998
scVI-batch	0.982
scVI-raw	0.972
scGPT zero-shot	0.951
CellPLM zero-shot	0.961
GenePT-w	0.838

Table S5. Graph Cell-Type Integration Local Inverse Simpson's Index (cLISI) scores across methods (listed as supplementary result due to the low variance of 0.001714)

Methods	w\ external knowledge	PCC (↑)	SCC (↑)	JSD (↓)	RMSE (↓)
sciLaMA-GenePT	√	0.220 ± 0.029	0.214 ± 0.031	0.280 ± 0.009	1.243 ± 0.023
sciLaMA-CellPLM	√	0.222 ± 0.027	0.218 ± 0.028	0.286 ± 0.009	1.242 ± 0.022
sciLaMA-ProtTrans	√	0.218 ± 0.026	0.211 ± 0.028	0.283 ± 0.009	1.246 ± 0.021
sciLaMA-ChatGPT	√	0.219 ± 0.027	0.217 ± 0.027	0.282 ± 0.009	1.244 ± 0.022
sciLaMA-scGPT	√	0.219 ± 0.027	0.217 ± 0.027	0.285 ± 0.009	1.244 ± 0.022
sciLaMA-ESM	√	0.233 ± 0.026	0.227 ± 0.027	0.282 ± 0.009	1.233 ± 0.022
sciLaMA (s.i.)	×	0.202 ± 0.027	0.212 ± 0.025	0.286 ± 0.009	1.258 ± 0.022
sciLaMA (random)	×	0.051 ± 0.027	0.049 ± 0.031	0.289 ± 0.009	1.374 ± 0.020
sciLaMA (shuffled)	×	0.056 ± 0.036	0.043 ± 0.037	0.288 ± 0.009	1.366 ± 0.027

Table S6. Evaluation of gene expression imputation performance on spatial transcriptomics data across variants of sciLaMA models

Methods	w/ external knowledge	Davies-Bouldin Index (↓)	Calinski-Harabasz Score (↑)
sciLaMA-GenePT	√	0.852	16.376
sciLaMA-CellPLM	√	0.727	19.610
sciLaMA-ProtTrans	√	0.802	19.947
sciLaMA-ChatGPT	√	0.874	16.522
sciLaMA-scGPT	√	0.780	17.973
sciLaMA-ESM	√	0.780	16.920
sciLaMA (s.i.)	×	0.977	13.087

Table S7. Clustering performance comparison for marker gene identification across variants of sciLaMA models

bioRxiv preprint doi: https://doi.org/10.1101/2025.01.28.635153; this version posted May 29, 2025. The copyright holder for this preprint (which was not certified by peer review) is the author/funder, who has granted bioRxiv a license to display the preprint in perpetuity. It is made available under aCC-BY 4.0 International license.

γ	ARI mean	ARI std	NMI mean	NMI std	ASW mean	ASW std	cLISI mean	cLISI std
0	0.464	0.371	0.513	0.390	0.589	0.087	0.881	0.170
0.01	0.582	0.021	0.734	0.022	0.645	0.010	0.990	0.002
0.05	0.665	0.114	0.763	0.041	0.654	0.005	0.991	0.002
0.1	0.634	0.088	0.751	0.023	0.658	0.012	0.992	0.002
0.25	0.581	0.024	0.743	0.013	0.655	0.006	0.990	0.002
0.5	0.592	0.010	0.747	0.004	0.658	0.012	0.993	0.001
0.75	0.590	0.015	0.748	0.013	0.656	0.012	0.992	0.002
1	0.647	0.107	0.762	0.038	0.651	0.025	0.993	0.003

Table S8. Effect of scalar γ on clustering performance across multiple metrics.

K Latent dim	ARI mean	ARI std	NMI mean	NMI std	ASW mean	ASW std	cLISI mean	cLISI std
10	0.651	0.110	0.756	0.042	0.654	0.005	0.991	0.002
20	0.627	0.082	0.761	0.025	0.631	0.015	0.991	0.000
30	0.583	0.017	0.742	0.022	0.633	0.014	0.991	0.001
40	0.680	0.114	0.771	0.039	0.637	0.009	0.991	0.002
50	0.606	0.085	0.743	0.023	0.631	0.010	0.990	0.002
60	0.649	0.100	0.757	0.030	0.631	0.015	0.990	0.002
70	0.590	0.037	0.738	0.017	0.635	0.009	0.991	0.001
80	0.649	0.095	0.753	0.020	0.632	0.009	0.991	0.001
90	0.656	0.102	0.756	0.030	0.637	0.008	0.991	0.001
100	0.641	0.108	0.751	0.027	0.635	0.006	0.991	0.002

Table S9. Effect of varying latent dimension K on clustering performance across multiple metrics.

bioRxiv preprint doi: https://doi.org/10.1101/2025.01.28.635153; this version posted May 29, 2025. The copyright holder for this preprint (which was not certified by peer review) is the author/funder, who has granted bioRxiv a license to display the preprint in perpetuity. It is made available under aCC-BY 4.0 International license.

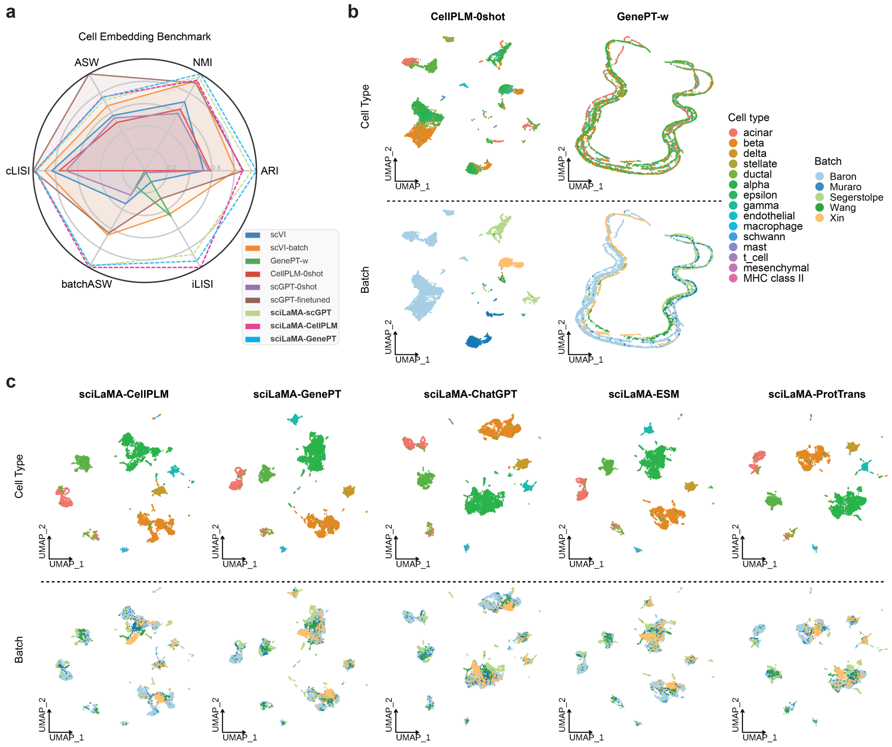

Figure S2. **Benchmark of cell representation learning. (a)** Radar plot showing the performance across six established metrics, comparing single cell SOTA methods (scVI and scVI-batch), zero-shot models (GenePT-w, CellPLM, and scGPT), a fine-tuned model (scGPT), and comparable sciLaMA-based models (sciLaMA-GenePT/CellPLM/scGPT). **(b-c)** UMAP visualizations of cell embeddings derived from various models, with colors indicating cell types (top) and batch origins (bottom). (b) includes foundation models in zero-shot mode, while (c) presents sciLaMA-based models in additional to those from Figure 2c.

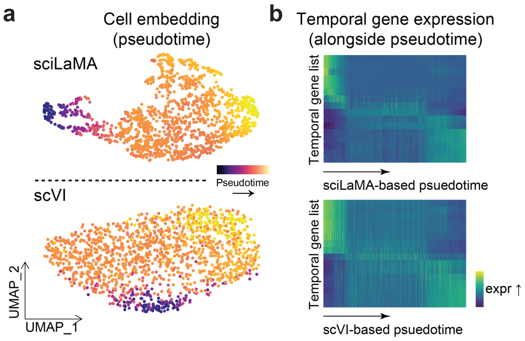

Figure S3. **Enhanced developmental cell trajectory analysis with sciLaMA. (a)** UMAP visualizations of cell embeddings from sciLaMA (top) and scVI (bottom) colored by inferred pseudotime via Palantir. **(b)** Heatmaps of dynamic gene expression changes by pseudotime (x-axis) with genes ordered by temporal specificity (y-axis). Top shows sciLaMA-based pseudotime, bottom shows scVI results.

bioRxiv preprint doi: https://doi.org/10.1101/2025.01.28.635153; this version posted May 29, 2025. The copyright holder for this preprint (which was not certified by peer review) is the author/funder, who has granted bioRxiv a license to display the preprint in perpetuity. It is made available under aCC-BY 4.0 International license.

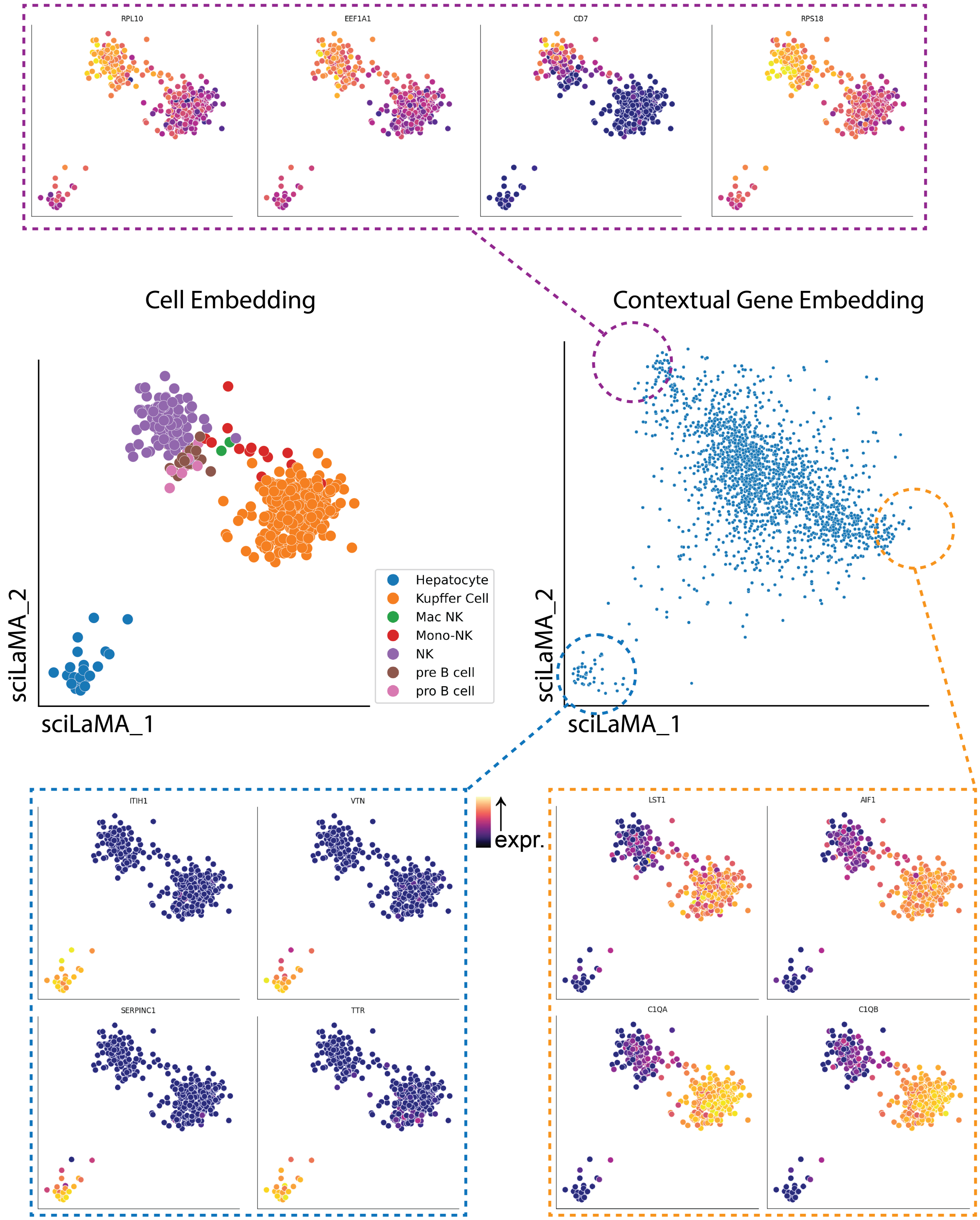

Figure S4. This visualization presents results from sciLaMA (with latent dimensionality K=2) trained on an exemplar fetal liver dataset (Choi et al., 2023). The cell embeddings (middle-left; dots represent individual cells) are colored according to annotated cell types, while the contextual gene embeddings (middle-right; dots represent individual genes) show corresponding embedding dimensions. The top and bottom panels illustrate cell embeddings colored by expression levels of genes sampled from distinct regions of the contextual gene embedding space. We quantitatively assessed sciLaMA's interpretability by computing feature attribution scores using Integrated Gradients applied to the pretrained cell VAE encoder. This resulted in a gene-by-latent-node attribution matrix, which we summarized into a single gene vector by aggregating absolute attribution scores. To facilitate comparison, we projected sciLaMA's contextual gene embeddings into a comparable vector form using vector normalization. The strong correlations (Pearson r = 0.43, Spearman ρ = 0.46) indicate that sciLaMA effectively captures key gene features in alignment with traditional stepwise attribution methods, yet does so within a more efficient, unified framework.
